# Characterization of Chinese *Haemophilus parasuis* Isolates by Traditional Serotyping and Molecular Serotyping Methods

**DOI:** 10.1371/journal.pone.0168903

**Published:** 2016-12-22

**Authors:** Lina Ma, Liyan Wang, Yuefeng Chu, Xuerui Li, Yujun Cui, Shengli Chen, Jianhua Zhou, Chunling Li, Zhongxin Lu, Jixing Liu, Yongsheng Liu

**Affiliations:** 1 State Key Laboratory of Veterinary Etiological Biology, Lanzhou Veterinary Research Institute, Chinese Academy of Agricultural Sciences, Lanzhou, Gansu, China; 2 State Key Laboratory of Pathogen and Biosecurity, Beijing Institute of Microbiology and Epidemiology, Beijing, China; 3 Institute of Animal Health, Guangdong Academy of Agricultural Sciences, Guangzhou, Guangdong, China; Public Health England, UNITED KINGDOM

## Abstract

*Haemophilus parasuis* is classified mainly through serotyping, but traditional serotyping always yields non-typable (NT) strains and unreliable results via cross-reactions. Here, we surveyed the serotype prevalence of Chinese *H*. *parasuis* isolates using traditional serotyping (gel immuno-diffusion test, GID) and molecular serotyping (multiplex PCR, mPCR). We also investigated why discrepant results between these methods were obtained, and investigated mPCR failure through whole-genome sequencing. Of the 100 isolate tested, 73 (73%) and 93 (93%) were serotyped by the GID test and mPCR, respectively, with a concordance rate of 66% (66/100). Additionally, mPCR reduced the number of NT isolates from 27 (27%) for the GID testing, to seven (7%). Eleven isolates were sequenced, including nine serotype-discrepant isolates from mPCR and GID typing (excluding strains that were NT by GID only) and two NT isolates from both methods, and their *in silico* serotypes were obtained from genome sequencing based on their capsule loci. The mPCR results were supported by the *in silico* serotyping of the seven serotype-discrepant isolates. The discrepant results and NT isolates determined by mPCR were attributed to deletions and unknown sequences in the serotype-specific region of each capsule locus. Compared with previous investigations, this study found a similar predominant serotype profile, but a different prevalence frequency for *H*. *parasuis*, and the five most prevalent serotypes or strain groups were serotypes 5, 4, NT, 7 and 13 for mPCR, and serotypes 5, NT, 4, 7 and 13/10/14 for GID. Additionally, serotype 7 was recognized as a principal serotype in this work.

## Introduction

*Haemophilus parasuis*, the causative agent of Glässer’s disease in pigs, has multiple clinical manifestations, including pneumonia, meningitis, arthritis, polyserositis, and septicemia [1–4]. Glässer’s disease outbreaks are seriously damaging to pigs and cause devastating economic losses to the swine industry worldwide, either on their own, or when co-infections with other swine pathogens occur [4–8]. Accurate serotype identification is critical for epidemiological investigations or vaccine selection studies in *H*. *parasuis* infections.

Generally, *H*. *parasuis* is classified by serotyping, and fifteen serotypes are recognized according to the current serotyping scheme [9]. Global serological surveys of *H*. *parasuis* have been carried out using traditional serotyping methods, and the prevalent serotypes are as follows: types 5, 4, 2, and 13 in Spain [10], 5, 4, and 13 in Denmark [11], 4, 5, 13, and 7 in North America [12], 1, 2, 4, 5, and 13 in the Netherlands [13], and 4, 5, 14, 13, and 2 in Brazil [14]. Epidemiological studies in China indicated that the prevalent serotypes were 4, 5, 13, 14 and 12 in 2005 [15] and 4, 5, 13, 15 and 2 in 2011 [16]. Regardless of whether *H*. *parasuis* is typed by the gel-immuno-diffusion (GID) test or the indirect haemagglutination assay (IHA), 10–40% of the non-typable (NT) strains [9–12, 15, 17–19] and frequent cross-reactions [12, 15, 16, 19, 20] were found in previous studies. Additionally, lack of the 15 serotype reference strains, difficulties in serovar-specific antigen and antiserum preparation [9, 17, 19], differences between antiserum batches [21], and the variable sensitivities of the detection methods [12, 21, 22] greatly decrease the capabilities of the traditional serotyping assays for typing *H*. *parasuis*.

Among the serotyping protocols available for *H*. *parasuis*, the capsular polysaccharide is assumed to be the dominant component of the serotyping antigen [9, 23–25]. The capsule loci for the 15 *H*. *parasuis* serotype reference strains have been annotated, and a strong correlation between the capsule locus type/*in silico* serotype and serotyping result was observed [26, 27]. Surprisingly, the capsule locus was also found in NT strains [27]. Therefore, the capsule locus offers a potential target for molecular serotyping of *H*. *parasuis*. Based on the concept of the capsule locus being responsible for the phenotype of the capsule, a multiplex PCR (mPCR) was developed by Howell et al. [28] for rapid molecular serotyping of *H*. *parasuis* [28]. All the isolates tested were typed by this method in that research study and a high concordance was gained between the mPCR and IHA results [28].

The aim of this study was to investigate *H*. *parasuis* serotype prevalence in Chinese pig herds. For this purpose, both mPCR and GID tests were performed, and whole-genome sequencing was used to validate the discrepant results between the mPCR and GID tests and to survey the cause of the mPCR serotyping failure. We found that the mPCR serotyping detection rate was superior to that of GID typing, and where discrepancies existed in the mPCR serotyping they were attributable to deletions and unknown sequences in the serotype-specific capsule locus region.

## Materials and Methods

### Ethics statement

This study was approved by the Animal Ethics Committee of Lanzhou Veterinary Research Institute, Chinese Academy of Agricultural Sciences (Permit No. LVRIAEC2007-003). All the experimental protocols in this study were conducted in strict accordance with the requirements of the Animal Ethics Procedures and Guidelines of the People’s Republic of China. All animals were humanely sacrificed under sodium pentobarbital anesthesia, and all efforts were made to minimize any suffering.

### Bacterial strains

A panel of 100 *H*. *parasuis* field isolates (S1 Table) was included in this study. The *H*. *parasuis* reference strains were kindly provided by Dr Patrick Blackall (Animal Research Institute, Queensland, Australia) and Dr Albert Rovira (Veterinary Diagnostic Laboratory, University of Minnesota, Minnesota, USA). All the isolates were collected from diseased pigs from Guangdong, Jiangsu, Shanghai, Qinghai, Gansu, Heilongjiang, and Jiangxi provinces of China between February 2007 and September 2014. The isolates were all characterized as *H*. *parasuis* in accordance with their colony characteristics [25], their Gram staining properties, nicotinamide adenine dinucleotide (NAD)-dependent tests [29, 30], and 16S RNA sequence identification [31]. The majority of the isolates originated from organs or tissues, including lung (n = 43), brain (n = 4), joint fluid (n = 2), cardiac blood (n = 10), lymph node (n = 3), pericardial effusion (n = 2), and abdominal effusion (n = 1), but 35 of the isolates lacked information about the isolation site.

### GID test

The serotypes of all the field isolates were identified using the GID test, as originally described by Morozumi and Nicolet [23]. Reference strain antisera were prepared as described previously [17] using cells grown overnight on tryptic soy agar (TSA, Becton, Dickinson and Company, Sparks, USA) supplemented with 5% horse serum and 10 μg/ml NAD. The serotyping antigen/heat-stable antigens from the field isolates were prepared by autoclaving at 121°C for 2 h as described by Morozumi and Nicolet [23]. The serotyping procedure was performed as described previously [19, 21]. The test was repeated once more if no definitive serotype was obtained for an isolate. NT strains were defined as isolates whose antigens did not react with antiserum against the 15 serotype reference strains.

### Multiplex PCR assay

The test procedure for the one-step mPCR was performed as previously described [28] with some modifications. Briefly, a loopful of bacteria from a pure culture plate was suspended in 30μl of UltraPure H_2_O. The mixture was boiled for 30 min, and the supernatant collected for each isolate after centrifugation at 4,000 × g for 1 min. A 1μl aliquot of genomic DNA for each sample was added to an mPCR mixture, and the 25μl total volume consisted of 12.5 μl premix Taq (Ex Taq version 2.0 plus dye), 0.5μl of primer mix (50 μM), 0.25μl DMSO (added at 1% of the total reaction volume), and 10.75μl of UltraPure H_2_O. All the samples were examined according to the serotype order, and the RNAse-free ddH_2_O and genomic DNA of the corresponding serotype reference strain were used as negative and positive controls, respectively. The dominant serotype 4 reference strain was used as the positive control for the NT strains. The mPCR was heated at 94°C for 5 min, followed by 30 cycles of 94°C for 30 s, 58°C for 30 s, 68°C for 60 s, and a final extension at 68°C for 5 min. The amplified products were electrophoresed in 2.0% agarose gels run in 1 x Tris-borate buffer with a Quick-Load® 100 bp DNA Ladder (New England BioLabs) as the molecular size standard. The procedure was repeated twice for each isolate.

### DNA extraction and genome sequencing

Eleven isolates were sequenced, including the 9 isolates with serotype discrepancies by mPCR and GID (excluding strains that were NT by GID only) and the 2 isolates that were NT by both methods. Genomic DNA was extracted from overnight cultures grown in supplemented TSB using a DNeasy Blood & Tissue Kit (Qiagen, Valencia, CA) according to the manufacturer’s instructions. The concentrations of the extracted genomic DNAs were measured using the Nanodrop 2000/2000C system (Thermo Scientific Company, Waltham, UK). All the isolates were sequenced on an Illumina HiSeq 2000 platform with a paired-end (PE) strategy. The SOAPdenovo assembly was performed using PE reads with quality filtering (Q20), first 5 nucleotides of the 5'-end, and adapter trimming. The average effective sequencing depth for all the isolates was 120-fold.

### Capsule locus identification and *in silico* serotype analysis

The *in silico* serotypes of the serotype-discrepant isolates and common NT isolates were determined by comparing their capsule contents and compositions with those of the reference strains. The capsule locus was identified for the 9 isolates with serotype discrepancies by mPCR and GID (excluding strains that were NT by GID only) and the 2 isolates that were NT by both methods according to a previous description [26] with some modifications. Briefly, the locus sequences were acquired by using the first gene (*funA*) and last gene (*iscR*) of the *H*. *parasuis* SH0165 capsule locus (GenBank accession No. CP001321.1) [32] as the query sequences. The gene name was determined for each coding sequence within the capsule locus by a nucleotide Basic Local Alignment Search Tool (BLASTn) interrogation of the NCBI database (https://www.ncbi.nlm.nih.gov/). The predicted gene names were recorded according to the highest matched nucleotide identity score. When more than one significant BLAST match sequence was found for a single isolate or various isolates, their identities were aligned further by BLASTn to determine whether the same sequence was obtained. Identical sequences were defined as described previously [27, 33], using a threshold of >80% nucleotide identity over >80% of coverage length. For simplicity, the capsule locus genes from each isolate were ordered from *funA* to *iscR*.

### Comparing the detection performances of mPCR and GID

The mPCR and GID performances were evaluated using the results from both of these analyses, and the *in silico* serotyping results. Because the *in silico* serotyping was 100% concordant with the mPCR results, it was considered to be a potential new gold standard for replacing traditional serotyping methods [28], and was used to validate the results of the above mentioned nine serotype-discrepant isolates. For the serotype-discrepant isolates from the GID and mPCR tests, the serotype category that agreed with the *in silico* serotyping was considered to be correct. The detection rate for the typable strains was calculated for the GID test and the mPCR assay, and the concordance between both methods was analyzed using the above information.

### Nucleotide sequence accession numbers

The genome sequences from this study were deposited in GenBank under the accession numbers MNAP00000000, MNAQ00000000, MNAR00000000, MNAS00000000, MNAT00000000, MNAU00000000, MNAV00000000, MNAW00000000, MNAX00000000, MNAY00000000, MNAZ00000000.

## Results

### Comparison of the prevalence and serotype profiles between mPCR and GID

**Fig 1:**
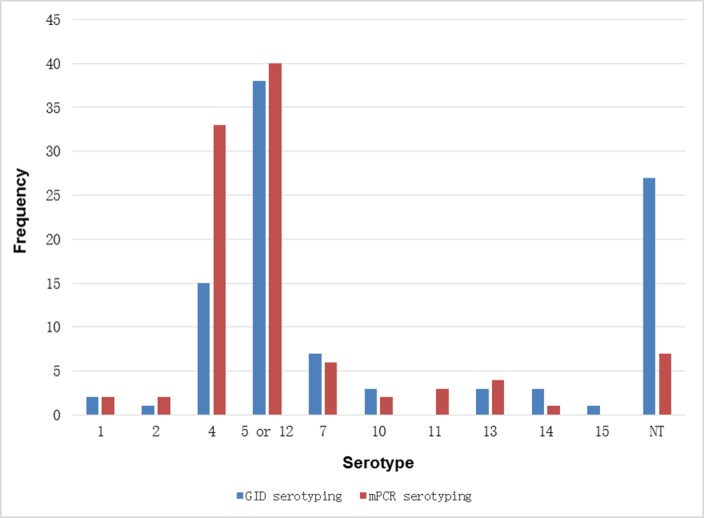
Serotype distribution of 100 Chinese isolates as determined by GID (blue) and mPCR (red).

A total of 100 Chinese *H*. *parasuis* clinical strains were tested using mPCR and GID methodologies. Of the 100 field isolates, 93 were typable, but the remaining seven were confirmed as NT by mPCR (Table 1). Regarding serotypes 5 and 12 as being the same serotype [28] (in this study, if not specified, serotype 5 refers to the serotype 5 and 12 combination), the most prevalent serotype identified by mPCR was serotype 5 (40% of isolates), followed by serotype 4 (33%), NT (7%), serotype 7 (6%), 13 (4%), 11 (3%), 1 (2%), 2 (2%), 10 (2%), and 14 (1%) (Fig 1). In contrast, only 73 field isolates were typable and 27 isolates were confirmed as NT strains by GID. The dominant serotype was serotype 5 (38%), followed by NT serotypes (27%), serotype 4 (15%), 7 (7%), 10 (3%), 13 (3%), 14 (3%), 1 (2%), 2 (1%), and 15 (1%) (Fig 1).

**Table 1:** Comparison of the results for mPCR and GID for 100 *H*. *parasuis* isolates.

Serotype by mPCR	Serotype by GID										
	1	2	4	5 or 12	7	10	13	14	15	NT	Total
**1**	1									1	2
**2**		1								1	2
**4**			15					2	1	15	33
**5 or 12**				38						2	40
**7**					4					2	6
**10**						1				1	2
**11**	1									2	3
**13**							3			1	4
**14**								1			1
**NT**					3	2				2	7
**Total**	2	1	15	38	7	3	3	3	1	27	100

Of the 73 serotyped isolates from the GID test, nine had results discrepant with mPCR. These serotype-discrepant isolates comprised a serotype 1 identified as a serotype 11 by mPCR, three serotype 7s identified as NT by mPCR, two serotype 10s identified as NT by mPCR, two serotype 14s identified as serotype 4 by mPCR, and a serotype 15 identified as serotype 4 by mPCR (Table 1). For the 73 typable isolates identified by GID, more than 87% (64/73) concordance was acquired between the mPCR and GID serotyping.

Of the 27 NT isolates identified by GID, 25 were identified as typable strains by mPCR and these were classified as the following 8 serotypes: serotype 1 (n = 1), 2 (n = 1), 4 (n = 15), 5 (n = 2), 7 (n = 2), 10 (n = 1), 11 (n = 2), and 13 (n = 1) (Table 1). H47 and K3, the remaining two NT isolates in the GID test, were also confirmed as NT by the mPCR method.

Although the distribution frequency of each serotype varied extremely between mPCR and GID, almost identical serotype profiles were identified by both methods. Additionally, mPCR and the GID showed nearly identical prevalences of the dominant serotypes or isolate group, and the first five most predominant groups, covering 90% of the total number of isolates, were completely identical for the two methods.

### *In silico* serotype analysis based on the capsule locus

Because nine isolates displayed different serotype results for GID and mPCR and two isolates were NT by both methods, the molecular basis of the inconsistent results and mPCR failures were investigated by whole-genome sequencing and analysis of the *in silico* serotypes obtained. Genome sequencing and assembly were finalized (Table 2), and the capsule loci were identified for the above mentioned serotype-discrepant isolates and NT isolates (Table 3) from the genome. *In silico* serotypes were obtained from the content and composition of each capsule locus. Compared with the capsule loci of the reference strains, these isolates displayed obvious deletions and/or unknown sequences (no significant similarity sequence in BLASTn search, NSSS) in their capsule loci. Deletions and NSSSs both occurred in the serotype-specific region of each capsule locus of these isolates. Furthermore, these deletions or NSSSs covered not only the serotype-specific gene position of the mPCR scheme, but also the signal regions of the *in silico* serotype analysis.

**Table 2:** General genome features of the sequenced *H*. *parasuis* isolates.

Strain	Accession No.	No.Scaffold	Size(Mb)	N50	GC Content
**H12**	MNAP00000000	139	2.19	41061	39.96
**H35**	MNAQ00000000	167	2.25	39573	39.91
**H36**	MNAR00000000	161	2.24	41150	39.9
**H38**	MNAW00000000	128	2.46	60021	39.54
**H39**	MNAX00000000	134	2.45	71054	39.52
**H47**	MNAS00000000	171	2.21	39317	39.86
**K3**	MNAY00000000	128	2.49	59987	39.48
**YT**	MNAU00000000	130	2.2	59301	39.85
**16**	MNAV00000000	140	2.2	55780	39.86
**HPS4**	MNAT00000000	112	2.21	60530	39.86
**HPS6**	MNAZ00000000	123	2.19	37861	39.85

**Table 3:** Summary of the capsule loci of the serotype-discrepant isolates and common NT isolates from the mPCR and GID tests.

Strain	serotype	Capsule composition																				
**SW124**^**a**^	**4**	*funA*	*neuA*	*wzx*	*mepA*	*gltF*	*wciP*	*gltG*	*lstB*	*lstA*	-^b^	-^b^	-^b^	-^b^	-^b^	-^b^	-^b^	-^b^	*wza*	*wzb*	*wzs*	*iscR*
**H12**	**4**	*funA*	*neuA*	*wzx*^f^	*mepA*	*gltF*	*wciP*	*gltG*	-^b^	*lstA*	-^b^	-^b^	-^b^	-^b^	-^b^	-^b^	-^b^	-^b^	*wza*	*wzb*	*wzs*	*iscR*
**H35**	**4**	*funA*	*neuA*	*wzx*^f^	*mepA*	*gltF*	*wciP*	*gltG*	-^b^	*lstA*	-^b^	-^b^	-^b^	-^b^	-^b^	-^b^	-^b^	-^b^	*wza*	*wzb*	*wzs*	*iscR*
**H36**	**4**	*funA*	*neuA*	*wzx*^f^	*mepA*	*gltF*	*wciP*	*gltG*	-^b^	*lstA*	-^b^	-^b^	-^b^	-^b^	-^b^	-^b^	-^b^	-^b^	*wza*	*wzb*	*wzs*	*iscR*
**H47**	**NT**	*funA*	*neuA*	*wzx*	NSSS1	NSSS2	NSSS3	NSSS4	*amsE*^c^	*lstA*	-^b^	-^b^	-^b^	-^b^	-^b^	-^b^	-^b^	-^b^	*wza*	*wzb*	*wzs*	*iscR*
**84–22113**^**a**^	**14**	*funA*	*mdhA*	*mviM*	*funAA*	*wbbJ*	*gstA*	*wzxB*	*funAB*	*funAC*	*gltQ*	*uaeA*	*wblI*	*wbgX*	*wbgY*	-^b^	-^b^	*capD*	*wza*	*wzb*	*wzs*	*iscR*
**84–15995**^**a**^	**15**	*funA*	*neuA*	*wzx*	*funI*	*funJ*	*gltR*	*gltE*	*gltM*	-^b^	-^b^	-^b^	*ndeB*	-^b^	*pilT*	*cap5M*	-^b^	*capD*	*wza*	*wzb*	*wzs*	*iscR*
**174**^**a**^	**7**	*funA*	*neuA*	*wzx*	*astA*	*funP*	*funQ*	*gltJ*	*cap5E*	*ndeA*	*naeA*	*gltA*	*ndeB*	-^b^	*pilT*	*cap5M*	-^b^	*capD*	*wza*	*wzb*	*wzs*	*iscR*
**HPS4**	**NT**	*funA*	*neuA*	*wzx*	*astA*	NSSS5	NSSS6	NSSS7	NSSS8	NSSS9	-^b^	-^b^	*ndeB*	*fun*^d^	*pilT*	*cap5M*	-^b^	*capD*	*wza*	*wzb*	*wzs*	*iscR*
**YT**	**NT**	*funA*	*neuA*	*wzx*	*astA*	-^b^	NSSS6	NSSS7	NSSS8	NSSS9	-^b^	-^b^	*ndeB*	*fun*^d^	*pilT*	*cap5M*	*amtA*	*capD*	*wza*	*wzb*	*wzs*	*iscR*
**16**^**e**^	**NT**	*funA*	*neuA*	*wzx*	-^b^	NSSS5	NSSS6	NSSS7	NSSS8	NSSS9	-^b^	-^b^	*ndeB*	*fun*^d^	*pilT*	*cap5M*	*amtA*	*capD*	*wza*	*wzb*	*wzs*	*iscR*
**C5**^**a**^	**8**	*funA*	*neuA*	*wzx*	*astA*	*gltH*	*adlA*	*scdA*	*funS*	*gltK*	-^b^	*wbgX*	*wbgY*	*funM*	*funN*	-^b^	-^b^	*capD*	*wza*	*wzb*	*wzs*	*iscR*
**H38**	**8**	*funA*	*neuA*	*wzx*	*astA*	*gltH*	*adlA*	NSSS10	*funS*	*gltK*	-^b^	*wbgX*	*wbgY*	*funM*	*funN*	-^b^	-^b^	*capD*	*wza*	*wzb*	*wzs*	*iscR*
**H39**	**8**	*funA*	*neuA*	*wzx*	*astA*	*gltH*	*adlA*	NSSS10	*funS*	*gltK*	-^b^	*wbgX*	*wbgY*	*funM*	*funN*	-^b^	-^b^	*capD*	*wza*	*wzb*	*wzs*	*iscR*
**K3**^**e**^	**1**	*funA*	*neuA*	*wzx*	*astA*	*gltH*	*adlA*	-^b^	*funS*	*gltK*	-^b^	*wbgX*	*wbgY*	*funM*	*funN*	-^b^	-^b^	*capD*	*wza*	*wzb*	*wzs*	*iscR*
**H555**^**a**^	**10**	*funA*	*neuA*	*wzx*	*astA*	*gltH*	*adlA*	*funX*	*funS*	*gltK*	-^b^	*wbgX*	*wbgY*	*funM*	-^b^	-^b^	-^b^	*capD*	*wza*	*wzb*	*wzs*	*iscR*
**No.4**^**a**^	**1**	*funA*	*funB*	*hydA*	*funC*	*gptA*	*wbuS*	*wzy*	*cap5E*	*ndeA*	*naeA*	*gltA*	*ndeB*	-^b^	-^b^	*cap5M*	-^b^	*capD*	*wza*	*wzb*	*wzs*	*iscR*
**H465**^**a**^	**11**	*funA*	*funB*	*hydA*	*funC*	*gptA*	*wbuS*	*wzy*	*cap5E*	*ndeA*	*naeA*	*gltO*	*bstA*	*actA*	*funY*	-^b^	*amtA*	*capD*	*wza*	*wzb*	*wzs*	*iscR*
**HPS6**	**11**	*funA*	*funB*	*hydA*	-^b^	*gptA*	-^b^	*wzy*	*cap5E*	*ndeA*	*naeA*	*gltO*	*bstA*	-^b^	-^b^	-^b^	*amtA*	*capD*	*wza*	*wzb*	*wzs*	*iscR*

All capsule loci are arranged in gene order from *funA* to *iscR*.

^a^serotype reference strain.

^b^ No gene exists in this position.

^c^*amsE*, the gene originating from *Mannheimia haemolytica* is annotated as pseudo in strains 89010807N and M42548. Here, H47 showed a truncated *amsE* with 75% nucleotide identity.

^d^*fun*, a sequence with unknown function. It shares 97% nucleotide identity and 100% coverage with the sequence (KF841370) encoding a hypothetical protein in the capsule gene locus of *Haemophilus parasuis* strain MN-H.

^e^the capsule loci for isolates 16 and K3 have not been assembled into a single continuous sequence.

^f^*wzx* is interrupted by two segments and is annotated as two copies of *wzx* in the whole-genome sequencing.

H12, H35, and H36 share some common capsule locus features with the serotype 4 reference strain, SW124, and their capsule loci obviously differ from those of the serotype 14 reference strain 22113 and the serotype 15 reference strain 15995 (Table 3). Compared with SW124, the three isolates only lacked the *lstB* gene in their capsule loci, so they were defined as belonging to capsule locus type 4 or *in silico* serotype 4.

H38, H39, and K3 share similar capsule loci with the C5 serotype 8 reference strain but these loci differed markedly from the H555 serotype 10 reference strain locus. Compared with C5, these three isolates lacked the *scdA* gene, but H38 and H39 share NSSS10 in this position (Table 3). Based on the capsule composition analysis, H38, H39 and K3 can be defined as *in silico* serotype 8.

Compared with the serotype 1 reference strain No.4, the capsule locus of HPS6 is more closely related to that of the serotype 11 reference strain H465 (Table 3). HPS6 shares *gltO*, *bstA* and *amtA* with H465; therefore, HPS6 is related to *in silico* serotype 11 based on its capsule composition.

HPS4, 16, and YT were identified separately as serotype 7 and NT strains by GID and mPCR; they share four NSSSs (NSSS6-NSSS9, with 100% nucleotide identity) and a sequence encoding a hypothetical protein named *fun*, as described previously [26] (Table 3). Moreover, HPS4 and 16 share NSSS5 with 100% nucleotide identity. Another gene, *amtA*, which is common in the capsule locus of the serotype 11 reference strain H465 [26], also appears in the capsule loci of YT and 16. In these two isolates the *funP*-*funQ*-*gltJ*-*cap5E*-*ndeA*-*naeA*-*gltA* gene cluster, which is part of the serotype-specific region for serotype 7 [26], is replaced by NSSSs; among these missing genes, *funQ* is the serotype-specific target gene for serotype 7 in the mPCR scheme [28]. Based on the capsule composition, HPS4, 16 and YT cannot be identified as a definite serotype in the *in silico* serotype analysis, so we defined them here as NT strains.

Compared with six other NT strains (YT, 16, HPS4, H38, H39 and K3) from the mPCR, H47 differs markedly in its capsule locus, which contains four continuous and totally different NSSSs (NSSS1-NSSS4) (Table 3). These NSSSs are also distinct from those of YT, 16, HPS4, H38 and H39. Although H47 contained the gene composition of *funA*-*neuA*-*wzx* and *lstA*-*wza*-*wzb*-*wzs*-*iscR*, it is still assumed to NT strain by the *in silico* analysis in this study because of the continuous NSSSs within its capsule locus.

### Comparison of the detection performance of mPCR and GID

The mPCR and GID detection rates were calculated using the data generated from GID, mPCR and the *in silico* serotype analysis. Of the 100 isolates we tested, 93 and 73 were identified as single serotypes by mPCR and GID, respectively. Compared with GID, the mPCR detection rate for serotyping *H*. *parasuis* isolates was 93%, a value higher than that of GID (73%).

Of the typable isolates tested by GID, nine showed discrepant results with mPCR. Taking the *in silico* serotype as the standard, the serotypes of seven serotype-discrepant isolates (H12, H35, H36, 16, YT, HPS4 and HPS6) from mPCR agreed well with the results of the *in silico* serotype analysis (Table 4). However, the *in silico* serotype analysis did not support any of the results from mPCR or GID for the remaining two serotype-discrepant isolates, H38 and H39 (Table 4). The concordance between the mPCR and GID test was 66% (66/100), including 64 typable isolates and two common NT strains by mPCR and the GID test.

**Table 4:** Test results comparison for GID, mPCR and *in silico* serotyping of the serotype-discrepant isolates.

Strain	Serotype by GID	Serotype by mPCR	*in silico* serotype
**HPS6**	serotype 1	serotype 11	serotype 11
**H12**	serotype 14	serotype 4	serotype 4
**H35**	serotype 15	serotype 4	serotype 4
**H36**	serotype 14	serotype 4	serotype 4
**H38**	serotype 10	NT	serotype 8
**H39**	serotype 10	NT	serotype 8
**16**	serotype 7	NT	NT
**YT**	serotype 7	NT	NT
**HPS4**	serotype 7	NT	NT

## Discussion

In this research, the results of mPCR and GID analyses produced nearly identical serotype profiles for the isolates. Considering serotypes 5 and 12 as the same serotype, the serotypes 5, 4, 7 and 13 are the most frequently detected serotypes in China, but the prevalence frequency for each serotype manifested obvious differences between the mPCR and GID tests. The results showed identical serotype profiles to those from studies in Denmark [11] and Canada [12], and an investigation of multinational samples [28]. Furthermore, studies performed in Germany [9], Spain [10], USA/Canada [17], Australia [34], China [15, 16] and Brazil [14] also had similar results. In all cases, serotype 4, 5 and 13 were the collectively predominant serotypes. Moreover, compared with previous reports from China, serotype 7 was the dominant serotype in this work.

High NT isolate rates were reported in all previous described studies, and the two most prevalent serotypes were 5 and 4. However, if the NT strains are included, they will become the third dominant *H*. *parasuis* isolate group in Germany [9], USA/Canada [17] and Denmark [11], and even exceed the number of serotype 4 and 5 isolates in some cases [10, 14]. When compared with serotyping by GID, mPCR substantially reduced the number of NT isolates from 27% to 7% in this work. The second dominant *H*. *parasuis* isolate group was changed from NT strains in the GID test to serotype 4 in the mPCR test. The number of NT isolates played a key role in the serotype profile and prevalence order for *H*. *parasuis*. It is also worth noting that a defect in capsule expression can still be existed, even though a entire capsule locus may be present in the isolates. Consequently, it is very important for NT strains to identify whether they are capsulated-strains or non-capsulated strains.

Capsule locus analysis of the NT strains from mPCR revealed the presence of a single deletion or NSSS at the position of a serotype-specific gene adopted by the mPCR for K3, H38, and H39, and multiple NSSSs at the serotype-specific region within this locus for YT, 16, HPS4, and H47. It is clear that the deletion of the serotype-specific target and emergence of NSSS within the capsule locus produced NT isolates in the mPCR test and in the accompanying *in silico* serotype analysis. In previous research [28], the deletions and NSSSs identified also resulted in a lower concordance between the mPCR and *in silico* serotype analysis. Similarly, insertions and deletions also caused discrepancies between the phenotypic and genotypic serotyping of *Shigella flexneri* [33]. To some extent, finding deletions and NSSSs in *H*. *parasuis* DNA probably indicates unstable serotype-specific regions within its capsule locus.

Overall, the principal serotype profile from mPCR and GID in our research was the same or similar to profiles of most other previous reports. As a genotypic serotyping method, mPCR is superior to phenotypic serotyping based on GID. In terms of the improved detection rate for typable isolates, the stability of the clinical test, and the compatibility of the results between different laboratories, mPCR will be a valuable alternative to the traditional serotype methods used for typing field isolates of *H*. *parasuis*. Investigation of the capsule expression and capsule structures are now required for exploring the origins of NT strains. Additionally, efforts should also be directed in future towards searching for more stable serotype-specific genes to remove the adverse impact of deletions and NSSSs in the mPCR test.

## Supporting Information

S1 Fig Band patterns of the molecular serotyping PCR for all 15 serotypes reference strains and part of isolates. M denotes Quick-load 100bp DNA Ladder (New England Biolabs Inc., USA). Lane 1: H_2_O (blank control). Lane 2: No.4 (serotype 1 reference strain). Lane 3: qixian. Lane 4: SW140 (serotype 2 reference strain). Lane 5: 211/212. Lane 6: SW114 (serotype 3 reference strain). Lane 7: SW124 (serotype 4 reference strain). Lane 8: H12. Lane 9: H23. Lane 10: H24. Lane 11: H25. Lane 12: H35. Lane 13: H36. Lane 14: H44. Lane 15: Nagasaki (serotype 5 reference strain). Lane 16: W1. Lane 17: ZX. Lane 18: H15. Lane 19: H17. Lane 20: H45. Lane 21: H46. Lane 22: 131 (serotype 6 reference strain). Lane 23: C5 (serotype 8 reference strain). Lane 24: D74 (serotype 9 reference strain). Lane 25: 174 ((serotype 7 reference strain)). Lane 26: H19. Lane 27: HE. Lane 28: HM. Lane 29: H555 (serotype 10 reference strain). Lane 30: H49. Lane 31: H465 (serotype 11 reference strain). Lane 32: HPS6. Lane 33: ST. Lane 34: H425 (serotype 12 reference strain). Lane 35: YZ-12. Lane 36: 84–17975 (serotype 13 reference strain). Lane 37: YZ-13. Lane 38: 84–22113 (serotype 14 reference strain). Lane 39: FS2. Lane 40: 84–15995 (serotype 15 reference strain). Lane 41: H38. Lane 42: H39. Lane 43: K3. Lane 44: 16. Lane 45: HPS4. Lane 46:YT. Lane 47: H47.
(TIF)

S1 Table Description of *H.parasuis* reference strains and isolates included in this study. (DOC)

## References

[1] Biberstein EL, White DC. A proposal for the establishment of two new Haemophilus species. J Med Microbiol. 1969; 2: 75–78. 10.1099/00222615-2-1-75 5821848

[2] Smart NLN, Miniats OP, Rosendal S, Friendship RRM, Miniats P. Glasser’s disease and prevalence of subclinical infection with *Haemophilus parasuis* in swine in southern Ontario. Can Vet J. 1989; 30: 339–343. 17423292 PMC1681197

[3] Aragon V, Cerdà-Cuéllar M, Fraile L, Mombarg M, Nofrarías M, Olvera A, et al Correlation between clinico-pathological outcome and typing of *Haemophilus parasuis* field strains. Vet Microbiol. 2010; 142: 387–93. 10.1016/j.vetmic.2009.10.025 19945233

[4] Oliveira S, Pijoan C. *Haemophilus parasuis*: new trends on diagnosis, epidemiology and control.Vet Microbiol. 2004; 99: 1–12. 10.1016/j.vetmic.2003.12.001 15019107

[5] Kim J, Chung H, Jung T, Cho W, Choi C, Chae C. Postweaning multisystemic wasting syndrome of pigs in Korea: prevalence, microscopic lesions an coexisting microorganisms. J Vet Sci. 2002; 64: 57–62. 10.1292/jvms.64.57 11853147

[6] Brockmeier SL, Halbur PG, Thacker EL. Porcine Respiratory Disease Complex In: Brogden K, Guthmiller J, editors. Polymicrobial Diseases. Washington: ASM Press; 2002 Pp. 231–238. 21735561

[7] Yu J, Wu J, Zhang Y, Guo L, Cong X, Du Y, et al Concurrent highly pathogenic porcine reproductive and respiratory syndrome virus infection accelerates *Haemophilus parasuis* infection in conventional pigs. Vet Microbiol. 2012; 158: 316–321. 10.1016/j.vetmic.2012.03.001 22460022

[8] Palzer A, Haedke K, Heinritzi K, Zoels S, Ladinig A, Ritzmann M. Associations among *Haemophilus parasuis*, *Mycoplasma hyorhinis*, and porcine reproductive and respiratory syndrome virus infections in pigs with polyserositis. Can Vet J. 2015; 56: 285–287. 25750450 PMC4327143

[9] Kielstein P, Rapp-Gabrielson VJ. Designation of 15 serovars of *Haemophilus parasuis* on the basis of immunodiffusion using heat-stable antigen extracts. J Clin Microbiol. 1992; 30: 862–865. 1572971 10.1128/jcm.30.4.862-865.1992 PMC265175

[10] Rúbies X, Kielstein P, Costa L, Riera P, Artigas C, Espuña E. Prevalence of *Haemophilus parasuis* serovars isolated in Spain from 1993 to 1997. Vet Microbiol. 1999; 66: 245–248. 10227126 10.1016/s0378-1135(99)00007-3

[11] Angen O, Svensmark B, Mittal KR. Serological characterization of Danish *Haemophilus parasuis* isolates. Vet Microbiol. 2004; 103: 255–258. 10.1016/j.vetmic.2004.07.013 15504597

[12] Tadjine M, Mittal KR, Bourdon S, Gottschalk M. Development of a new serological test for serotyping *Haemophilus parasuis* isolates and determination of their prevalence in North America. J Clin Microbiol. 2004; 42: 839–840. 10.1128/JCM.42.2.839-840.2004 14766867 PMC344452

[13] Dijkman R, Wellenberg GJ, van der Heijden HM, Peerboom R, Olvera A, Rothkamp A, et al Analyses of Dutch *Haemophilus parasuis* isolates by serotyping, genotyping by ERIC-PCR and Hsp60 sequences and the presence of the virulence associated trimeric autotransporters marker. Res Vet Sci. 2012; 93(2):589–595. 10.1016/j.rvsc.2011.10.013 22119186

[14] Castilla KS, de Gobbi DDS, Moreno LZ, Paixão R, Coutinho TA, dos Santos JL, et al Characterization of *Haemophilus parasuis* isolated from Brazilian swine through serotyping, FLP and PFGE. Res Vet Sci. 2012; 92:366–371. 10.1016/j.rvsc.2011.04.006 21529864

[15] Cai X, Chen H, Blackall PJ, Yin Z, Wang L, Liu Z, et al Serological characterization of *Haemophilus parasuis* isolates from China. Vet Microbiol. 2005; 111: 231–236. 10.1016/j.vetmic.2005.07.007 16271834

[16] Zhang J, Xu C, Guo L, Ke B, Ke C, Zhang B, et al A rapid pulsed-field gel electrophoresis method of genotyping *Haemophilus parasuis* isolates. Lett Appl Microbiol. 2011; 52: 589–95. 10.1111/j.1472-765X.2011.03048.x 21507027

[17] Rapp-Gabrielson VJ, Gabrielson DA. Prevalence of *Haemophilus parasuis* serovars among isolates from swine. Am J Vet Res. 1992; 53: 659–664. 1524289

[18] Oliveira S, Blackall PJ, Pijoan C. Characterization of the diversity of *Haemophilus parasuis* field isolates by use of serotyping and genotyping. Am J Vet Res. 2003; 64: 435–442. 12693533 10.2460/ajvr.2003.64.435

[19] Rafiee M, Blackall PJ. Establishment, validation and use of Kielstein-Rapp-Gabrielson serotyping scheme for *Haemophilus parasuis*. Aus Vet J. 2000; 78: 172–174. 10.1111/j.1751-0813.2000.tb10586.x 10860155

[20] Turni C, Blackall PJ. Serovar profiling of *Haemophilus parasuis* on Australian farms by sampling live pigs. Aust Vet J. 2010; 88: 255–9. 10.1111/j.1751-0813.2010.00592.x 20579030

[21] Turni C, Blackall PJ. Comparison of the indirect haemagglutination and gel diffusion test for serotyping *Haemophilus parasuis*. Vet Microbiol. 2005; 106: 145–151. 10.1016/j.vetmic.2004.12.019 15737484

[22] Del Río ML, Gutiérrez CB, Rodríguez Ferri EF. Value of indirect hemagglutination and coagglutination tests for serotyping *Haemophilus parasuis*. J Clin Microbiol. 2003; 41: 880–882. 10.1128/JCM.41.2.880-882.2003 12574306 PMC149707

[23] Morozumi T, Nicolet J. Some antigenic properties of *Haemophilus parasuis* and a proposal for serological classification. J Clin Microbiol. 1986; 23: 1022–1025. 3086374 10.1128/jcm.23.6.1022-1025.1986 PMC268784

[24] Kielstein P, Rosner H, Muller W. Typing of heat-stable soluble *Haemophilus parasuis* antigen by means of agar gel precipitation and the dot-blot procedure. J Vet Med B. 1991; 38: 315–320. 10.1111/j.1439-0450.1991.tb00877.x 1832258

[25] Morozumi T, Nicolet J. Morphological variations of *Haemophilus parasuis* strains. J Clin Microbiol. 1986; 23: 138–142. 3700597 10.1128/jcm.23.1.138-142.1986 PMC268588

[26] Howell KJ, Weinert LA, Luan S-L, Peters SE, Chaudhuri RR, Harris D, et al Gene content and diversity of the loci encoding biosynthesis of capsular polysaccharides of the fifteen serovar reference strains of *Haemophilus parasuis*. J Bacteriol. 2013; 195: 4264–4273. 10.1128/JB.00471-13 23873912 PMC3754760

[27] Howell KJ, Weinert LA, Chaudhuri RR, Luan S, Peters SE, Corander J, et al The use of genome wide association methods to investigate pathogenicity, population structure and serovar in *Haemophilus parasuis*. BMC Genomics. 2014;15: 1179 10.1186/1471-2164-15-1179 25539682 PMC4532294

[28] Howell KJ, Peters SE, Wang J, Hernandez-Garcia J, Weinert LA, Luan SL, et al Development of a multiplex PCR assay for rapid molecular serotyping of *Haemophilus parasuis*. J Clin Microbiol. 2015; 53: 3812–3821. 10.1128/JCM.01991-15 26424843 PMC4652097

[29] Møller K, Kilian M. V factor-dependent members of the family Pasteurellaceae in the porcine upper respiratory tract. J Clin Microbiol. 1990; 28: 2711–2716. 2280002 10.1128/jcm.28.12.2711-2716.1990 PMC268260

[30] Møller K, Andersen LV, Christensen G, Kilian M. Optimization of the detection of NAD dependent Pasteurellaceae from the respiratory tract of slaughter-house pigs. Vet Microbiol. 1993; 36: 261–271. 8273273 10.1016/0378-1135(93)90093-m

[31] Angen O, Oliveira S, Ahrens P, Svensmark B, Leser TD. Development of an improved species specific PCR test for detection of *Haemophilus parasuis*.Vet Microbiol. 2007; 119: 266–276. 10.1016/j.vetmic.2006.10.008 17113728

[32] Yue M, Yang F, Yang J, Bei W, Cai X, Chen L, et al Complete genome sequence of *Haemophilus parasuis* SH0165. J Bacteriol. 2009; 191: 1359–1360. 10.1128/JB.01682-08 19074396 PMC2632009

[33] Gentle A, Ashton PM, Dallman TJ, Jenkins C. Evaluation of molecular methods for serotyping *Shigella fleneri*. J Clin Microbiol. 2016; 54: 1456–1461. 10.1128/JCM.03386-15 26984974 PMC4879286

[34] Blackall PJ, Rapp-Gabrielson V, Hampson DJ. Serological characterization of *Haemophilus parasuis* isolates from Australian pigs. Aust Vet J. 1996; 73: 93–95. 8660221 10.1111/j.1751-0813.1996.tb09984.x

