# Supplementary figures and images for: Characterization of Chinese Haemophilus parasuis Isolates by Traditional Serotyping and Molecular Serotyping Methods
Source: PLoS One. 2016 Dec 22;11(12):e0168903. doi: 10.1371/journal.pone.0168903 (PMC5179118; doi:10.1371/journal.pone.0168903)

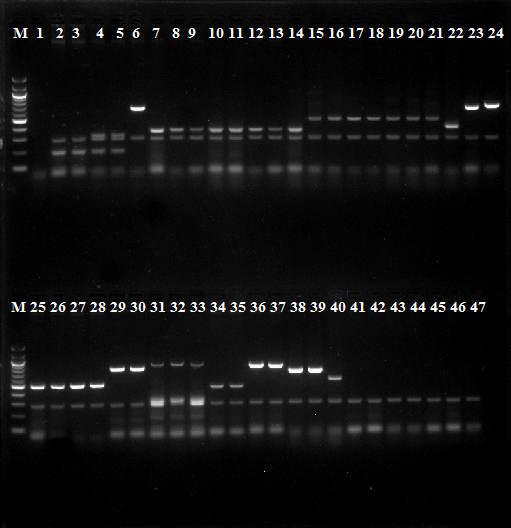

Supplement: S1 Fig — M denotes Quick-load 100bp DNA Ladder (New England Biolabs Inc., USA). Lane 1: H2O (blank control). Lane 2: No.4 (serotype 1 reference strain). Lane 3: qixian. Lane 4: SW140 (serotype 2 reference strain). Lane 5: 211/212. Lane 6: SW114 (serotype 3 reference strain). Lane 7: SW124 (serotype 4 reference strain). Lane 8: H12. Lane 9: H23. Lane 10: H24. Lane 11: H25. Lane 12: H35. Lane 13: H36. Lane 14: H44. Lane 15: Nagasaki (serotype 5 reference strain). Lane 16: W1. Lane 17: ZX. Lane 18: H15. Lane 19: H17. Lane 20: H45. Lane 21: H46. Lane 22: 131 (serotype 6 reference strain). Lane 23: C5 (serotype 8 reference strain). Lane 24: D74 (serotype 9 reference strain). Lane 25: 174 ((serotype 7 reference strain)). Lane 26: H19. Lane 27: HE. Lane 28: HM. Lane 29: H555 (serotype 10 reference strain). Lane 30: H49. Lane 31: H465 (serotype 11 reference strain). Lane 32: HPS6. Lane 33: ST. Lane 34: H425 (serotype 12 reference strain). Lane 35: YZ-12. Lane 36: 84–17975 (serotype 13 reference strain). Lane 37: YZ-13. Lane 38: 84–22113 (serotype 14 reference strain). Lane 39: FS2. Lane 40: 84–15995 (serotype 15 reference strain). Lane 41: H38. Lane 42: H39. Lane 43: K3. Lane 44: 16. Lane 45: HPS4. Lane 46:YT. Lane 47: H47. (TIF) [file pone.0168903.s001.tif]
