# Supplementary material for: Characterization of Chinese Haemophilus parasuis Isolates by Traditional Serotyping and Molecular Serotyping Methods
Source: PLoS One. 2016 Dec 22;11(12):e0168903. doi: 10.1371/journal.pone.0168903 (PMC5179118; doi:10.1371/journal.pone.0168903)
Supplement: S1 Table — (DOC) [file pone.0168903.s002.doc]

Table S1. Description of *H. parasuis* reference strains and isolates included in this study.

| **Strain** | **Country/region** | **Isolation site** | **Isolation/collection time** |
| --- | --- | --- | --- |
| No.4^a^ | Japan | nose | unknown |
| SW140^a^ | Japan | nose | unknown |
| SW114^a^ | Japan | nose | unknown |
| SW124^a^ | Japan | nose | unknown |
| Nagasaki^a^ | Japan | meninges | unknown |
| 131^a^ | Switzerland | nose | unknown |
| 174^a^ | Switzerland | nose | unknown |
| C5^a^ | Sweden | unknown | unknown |
| D74^a^ | Sweden | unknown | unknown |
| H555^a^ | Germany | nose | unknown |
| H465^a^ | Germany | trachea | unknown |
| H425^a^ | Germany | lung | unknown |
| 84-17975^a^ | United States | lung | unknown |
| 84-22113^a^ | United States | joint | unknown |
| 84-15995^a^ | United States | lung | unknown |
| 31 | Jiangsu | Unknown | 2007 |
| 3031 | Jiangsu | Unknown | 2007 |
| 3032-1 | Jiangsu | Unknown | 2007 |
| 3033 | Jiangsu | Unknown | 2007 |
| 001 | Jiangsu | Unknown | 2007 |
| HPS-4-30 | Jiangsu | Unknown | 2007 |
| 30 | Jiangsu | Unknown | 2007 |
| HPS30 | Jiangsu | Unknown | 2007 |
| 130 | Jiangsu | Unknown | 2007 |
| qixian | Shanghai | Lung | 2007 |
| jx2 | Jiangxi | Unknown | 2007 |
| jx4 | Jiangxi | Unknown | 2007 |
| jx5 | Jiangxi | Unknown | 2007 |
| jx7 | Jiangxi | Unknown | 2007 |
| H04 | Guangdong | Unknown | 2007 |
| H08 | Guangdong | Brain | 2007 |
| H9 | Guangdong | Lung | 2007 |
| H10 | Guangdong | Brain | 2007 |
| H11 | Guangdong | Lung | 2007 |
| H12 | Guangdong | lymph node | 2007 |
| H13 | Guangdong | Lung | 2007 |
| H14 | Guangdong | Lung | 2007 |
| H15 | Guangdong | Cardiac blood | 2007 |
| H17 | Guangdong | Lung | 2007 |
| H19 | Guangdong | Cardiac blood | 2007 |
| H20 | Guangdong | Cardiac blood | 2007 |
| H21 | Guangdong | Lung | 2007 |
| H22 | Guangdong | Cardiac blood | 2008 |
| H23 | Guangdong | Lung | 2008 |
| H24 | Guangdong | Lung | 2008 |
| H25 | Guangdong | Abdominal effusion | 2008 |
| H26-1 | Guangdong | Cardiac blood | 2008 |
| H27 | Guangdong | Joint fluid | 2008 |
| H29 | Guangdong | Lung | 2008 |
| H30 | Guangdong | Cardiac blood | 2008 |
| H31 | Guangdong | Lung | 2008 |
| H32 | Guangdong | Cardiac blood | 2008 |
| H33-1 | Guangdong | Cardiac blood | 2008 |
| H34 | Guangdong | Lung | 2008 |
| H35 | Guangdong | Cardiac blood | 2008 |
| H36 | Guangdong | Joint fluid | 2008 |
| H37 | Guangdong | lymph node | 2008 |
| H38 | Guangdong | Lung | 2008 |
| H39 | Guangdong | lymph node | 2008 |
| H40 | Guangdong | Lung | 2008 |
| H43 | Guangdong | Brain | 2008 |
| H44 | Guangdong | Lung | 2008 |
| H45 | Guangdong | Pericardial effusion | 2008 |
| H46 | Guangdong | Pericardial effusion | 2008 |
| H47 | Guangdong | Brain | 2008 |
| H48 | Guangdong | Cardiac blood | 2008 |
| H49 | Guangdong | Unknown | 2008 |
| H5R | Gansu | Lung | 2008 |
| ZH2 | Jiangsu | Lung | 2008 |
| ZH3 | Jiangsu | Lung | 2008 |
| 16 | Jiangsu | Lung | 2008 |
| D09 | Jiangsu | Unknown | 2008 |
| YT | Jiangsu | Lung | 2008 |
| 211/212 | Jiangsu | Lung | 2008 |
| 124 | Jiangsu | Unknown | 2008 |
| W1 | Jiangsu | Unknown | 2008 |
| 2x62x | Jingsu | Unknown | 2008 |
| 1117 | Qinghai | Lung | 2008 |
| HQ1111 | Qinghai | Unknown | 2008 |
| TC1 | Jiangsu | Unknown | 2009 |
| TC7 | Jiangsu | Lung | 2009 |
| K3 | Jiangsu | Lung | 2009 |
| IV-3 | Unknown | Unknown | 2009 |
| WX | Unknown | Unknown | 2009 |
| ZJ | Guangdong | Unknown | 2009 |
| DG | Guangdong | Unknown | 2009 |
| HM | Guangdong | Unknown | 2009 |
| ST | Guangdong | Unknown | 2009 |
| CS | Guangdong | Unknown | 2009 |
| HPS4 | Heilongjiang | Lung | 2009 |
| HPS6 | Heilongjiang | Lung | 2009 |
| ZX | Unknown | Unknown | 2009 |
| HPS7 | Heilongjiang | Lung | 2010 |
| Q7 | Heilongjiang | Lung | 2010 |
| FS1 | Guangdong | Lung | 2014 |
| FS2 | Guangdong | Lung | 2014 |
| FS3 | Guangdong | Lung | 2014 |
| FS4 | Guangdong | Lung | 2014 |
| FS5 | Guangdong | Lung | 2014 |
| FS6 | Guangdong | Lung | 2014 |
| FS7 | Guangdong | Lung | 2014 |
| FS8 | Guangdong | Lung | 2014 |
| FS9 | Guangdong | Lung | 2014 |
| FS10 | Guangdong | Lung | 2014 |
| FS12 | Guangdong | Lung | 2014 |
| FS13 | Guangdong | Lung | 2014 |
| FS15 | Guangdong | Lung | 2014 |
| FS16 | Guangdong | Lung | 2014 |
| L1 | Guangdong | Lung | 2014 |
| HE | Guangdong | Unknown | 2014 |
| HF | Guangdong | Unknown | 2014 |
| YZ-4 | Jiangsu | Unknown | 2014 |
| YZ-5 | Jiangsu | Unknown | 2014 |
| YZ-12 | Jiangsu | Unknown | 2014 |
| YZ-13 | Jiangsu | Unknown | 2014 |

^a^ the reference strains, originally published by Kielstein and Rapp-Gabrielson (1992).
